# Functional phosphoproteomic analysis of SMG1 in nonsense-mediated mRNA decay and DNA damage repair in cancer

**DOI:** 10.3389/fsysb.2026.1788470

**Published:** 2026-08-07

**Authors:** Apoorva K. A. Pai, Leona Dcunha, Athira Perunelly Gopalakrishnan, Samseera Ummar, Athira C. Rajeev, Rajesh Raju

**Affiliations:** Centre for Integrative Omics Data Science (CIODS), Yenepoya (Deemed to be University), Mangalore, Karnataka, India

**Keywords:** cancer, DNA damage response, non-sense mediated mRNA decay, phosphoproteomics, SMG1

## Abstract

**Introduction:**

SMG1, a phosphatidylinositol 3-kinase-related kinase (PIKK) family serine/threonine kinase, is a key regulator of nonsense-mediated mRNA decay (NMD) through phosphorylation of UPF1 and plays important roles in genome stability, p53 activation, and tumor suppression. Although 77 phosphorylation sites have been reported on SMG1, their regulatory significance has not been systematically characterized.

**Methods:**

A comprehensive meta-analysis of more than 3,800 PubMed-indexed human phosphoproteomic studies was performed to curate Class I SMG1 phosphosites. A total of 678 qualitative and 173 quantitative phosphoproteomic datasets were analyzed to identify predominant phosphorylation sites. Co-regulation, co-occurrence, motif enrichment, kinase prediction, and cancer phosphoproteomic analyses were conducted to investigate site-specific regulatory networks and biological functions.

**Results:**

Three C-terminal phosphosites (T3573, S3570, and S3556) emerged as the most frequently detected and dynamically regulated sites across datasets. Co-regulation network analysis indicated distinct functional associations for each site, with T3573 linked to cytoskeletal regulators, S3570 to RNA-processing factors, and S3556 to chromatin- and DNA damage-associated proteins. Co-occurrence analysis suggested that these phosphosites can be phosphorylated simultaneously, indicating coordinated regulation. Multiple core NMD components, including SMG7, UPF1, SMG9, CASC3, and CTIF, exhibited strong positive co-regulation with S3570 and/or S3556. In silico and peptide-array-based kinase predictions identified ATM, PRKDC (DNA-PKcs), and BRAF as candidate upstream kinases for S3556, linking SMG1 phosphorylation to DNA damage signaling. Motif and co-regulation analyses expanded the putative SMG1 substrate repertoire by identifying numerous [S/T]-Q-containing DNA damage response proteins, including BRCA1, RAD51AP1, NBN, PNKP, TP53BP1, and PBRM1. UALCAN analysis further revealed differential regulation of SMG1 phosphosites across multiple cancer types.

**Discussion:**

These findings identify SMG1 as a central phosphorylation hub integrating nonsense-mediated mRNA decay with genome maintenance pathways and provide the first systematic phosphosite-centric framework for understanding its regulation. The identified regulatory phosphosites, candidate upstream kinases, and phosphorylation networks establish a foundation for future mechanistic studies and support the potential of SMG1 phosphorylation as a diagnostic and therapeutic target in cancer.

## 1 Introduction

SMG1 is an atypical ∼410 kDa serine/threonine protein kinase belonging to the phosphatidylinositol 3-kinase-related kinase (PIKK) family. SMG1 has been implicated in cellular stress response pathways and is best characterized for its role in nonsense-mediated mRNA decay (NMD). In addition, it has been associated with processes such as telomere maintenance, hypoxia adaptation, and TNF-α-mediated apoptosis (McIlwain et al., 2010). Together with its regulatory partners SMG8 and SMG9, SMG1 forms the heterotrimeric SMG1C complex that regulates its kinase activity (Zhu et al., 2019).

Human SMG1 is a large protein of 3,661 amino acids (UniProt Q96Q15) encoded on chromosome 16p12.3. It is widely expressed, with highest levels in heart and skeletal muscle, and localizes to the nucleus, nucleoplasm, cytosol, and specialized structures such as chromatoid bodies (Brumbaugh et al., 2004). Structurally, SMG1 comprises an extended N-terminal HEAT-repeat solenoid, a central catalytic core (encompassing middle HEAT repeats, FAT domain, kinase domain, and FATC domain), and an N-terminal intrinsically disordered region that mediates interactions with the mRNA surveillance machinery (Brown et al., 2011). The kinase preferentially phosphorylates serine/threonine-glutamine (SQ) motifs preceded by hydrophobic residues in substrates such as UPF1, thereby initiating NMD and stabilizing p53 under genotoxic stress (Langer et al., 2020; Roberts et al., 2013). Recent cryo-EM structures have illuminated how the SMG1–SMG8–SMG9 complex recognizes substrates and how its insertion domain, stabilized by SMG8, enforces autoinhibition (Langer et al., 2021).

Emerging evidence positions SMG1 as a bonafide tumor suppressor in both solid tumors and hematological malignancies (Hojjatipour et al., 2022). Inactivation, often through promoter hypermethylation or other epigenetic mechanisms, has been documented in pancreatic cancer, nasopharyngeal carcinoma, acute myeloid leukemia, and certain breast cancer subtypes (Roberts et al., 2013). Its tumor-suppressive functions are thought to stem from its dual roles in DNA damage response and suppression of chronic inflammation (Ho et al., 2019). Pharmacological inhibition of SMG1 disrupts NMD, triggers endoplasmic reticulum stress and the unfolded protein response, and sensitizes cancer cells to apoptosis, as exemplified by the dual DNA-PK/mTOR inhibitor CC-115 in multiple myeloma (Leeksma et al., 2023). Moreover, SMG1 inhibition alters TP53 pre-mRNA splicing to favor β and γ isoforms without activating canonical p53α targets (McCann et al., 2023).

Despite extensive characterization of its domain architecture, substrate specificity, and biological roles, the global phosphorylation profile of SMG1 itself remains largely uncharted. In this study, we systematically mapped SMG1 phosphosites using large-scale phospho-proteomic datasets, revealing that most regulated sites reside outside the canonical structural domains. By analyzing co-regulation networks, we identified phosphosites on interacting partners, candidate upstream kinases, and downstream effectors that correlate with SMG1 phosphorylation status. Together, these findings provide new insight into the post-translational control of this atypical PIKK family tumor suppressor and its broader implications in cancer biology.

## 2 Materials and methods

### 2.1 Comprehensive screening of global phosphoproteome data and curation of SMG1 phosphopeptides

To comprehensively examine the phosphorylation landscape of SMG1, a systematic literature search was performed in PubMed using the keywords “phosphoproteomics” OR “phosphoproteome,” restricted to studies on human cell lines and excluding plant-model and review articles. The search retrieved over 3,800 candidate articles, which were screened by title and abstract and then by full text to identify mass spectrometry–based phosphoproteomic datasets reporting SMG1 phosphosites. Only high-quality datasets generated by mass spectrometry in human cell lines were retained, to ensure direct biological relevance and to exclude lower-confidence detection methods.

Through manual curation, datasets reporting Class-1 SMG1 phosphosites, defined by a localization probability of ≥75% and an Ascore greater than 13, were compiled, consistent with the threshold conventionally used to denote high-confidence, unambiguously localized phosphosites in shotgun phosphoproteomics. Dataset selection emphasized studies reporting SMG1 phosphopeptides generated using LysC and/or trypsin digestion, the most widely applied proteolytic strategies in phosphoproteomic workflows, following the same curation framework used in our companion analyses (Dcunha et al., 2025; Chakraborty et al., 2025; Gopalakrishnan et al., 2025).

To systematically structure the curated data, datasets were divided into two categories:

- Profile datasets, which report test and control conditions independently and provide a broad, qualitative overview of detected phosphosites.
- Differential datasets, which quantify changes in phosphosite abundance between a defined test condition and its corresponding control, thereby revealing phosphorylation dynamics under specific biological contexts.

For differential datasets, Class-1 phosphosites were classified as upregulated when showing a fold change ≥1.3, and downregulated when the fold change was ≤0.76, with statistical significance assessed according to the criteria specified in each original study (p < 0.05). No additional fold-change recalculation or p-value correction was applied beyond what was reported in the source publication, given the heterogeneity of quantification platforms across studies. These thresholds were applied to ensure that only biologically meaningful phosphorylation events were included in downstream analysis.

### 2.2 Protein and phosphosite mapping pipeline

A custom mapping pipeline was developed for this study to ensure consistency and accuracy of protein and phosphosite identifiers across the assembled datasets. At the protein level, all identified proteins were standardized to their official gene symbols using the HUGO Gene Nomenclature Committee (HGNC) database (Bruford et al., 2020), using the most up-to-date HGNC release available at the time of curation to harmonize naming across datasets that often varied in annotation conventions.

At the phosphosite level, each site was mapped to its corresponding UniProt accession number using the UniProt database release downloaded on 13 May 2023 (UniProt Consortium, 2023). The mapping pipeline extracted phosphosite-specific information, residue type (serine, threonine, or tyrosine), sequence position, and flanking sequence, from each dataset and aligned this information to the corresponding canonical UniProt protein sequence. Ambiguities arising from non-canonical isoform usage or inconsistent residue numbering across source studies were identified and resolved through validation checks, using flanking-sequence comparison to confirm correct positional mapping, in order to ensure high mapping accuracy and reproducibility.

Each dataset was further annotated with standardized metadata tags describing its biological system and experimental conditions (e.g., cell line, stimulus, genetic perturbation, treatment duration), following the structured condition-coding format detailed in Sanjeev et al. (2024) (Sanjeev et al., 2024), to enable efficient and consistent cross-study categorization and interpretation.

### 2.3 Identification of predominant phosphorylation sites of SMG1

Phosphoproteome profiling datasets derived from human cell lines were used to identify Class-1 SMG1 phosphosites. For each phosphosite, two frequency metrics were determined: (i) its frequency of appearance across profile datasets, and (ii) its frequency of differential regulation across differential datasets. Phosphosites were ranked independently by each metric. Predominant SMG1 phosphosites were defined as those detected in, or differentially regulated in, over 50% of the respective profile and differential datasets.

### 2.4 Investigation of protein phosphosites co-differentially regulated with predominant phosphorylation sites of SMG1

To identify phosphosites in other proteins (PsOPs) whose expression correlated, positively or negatively, with the predominant SMG1 phosphosites, each quantitative differential dataset was analyzed independently. Owing to the large number of datasets spanning diverse biological systems, experimental conditions, and analytical platforms, reprocessing of raw mass spectrometry data was not feasible; co-differential regulation was instead assessed directly from the previously reported, study-level differential calls, following the same general methodological framework applied in other analyses from our lab.

For each predominant SMG1 phosphosite (denoted “s”), datasets in which it was differentially regulated were used to classify co-detected phosphosites in other proteins (denoted “o”) according to their own regulation direction, generating four possible joint categories:

- UoUs- PsOP upregulated when the SMG1 site was upregulated.
- DoDs- PsOP downregulated when the SMG1 site was downregulated.
- DoUs- PsOP downregulated when the SMG1 site was upregulated.
- UoDs- PsOP upregulated when the SMG1 site was downregulated.

Of these, UoUs and DoDs were classified as positively co-regulated events, whereas DoUs and UoDs were classified as negatively co-regulated events relative to the corresponding SMG1 phosphosite.

#### 2.4.1 Statistical filtering

To assess the statistical robustness of each candidate SMG1 site- PsOP relationship, a one-sided Fisher’s Exact Test (FET) was applied to a contingency table constructed. Candidate PsOPs were retained only if they satisfied all the following criteria simultaneously:

1. FET p-value <0.05.
2. A co-regulation ratio of at least 10%, calculated as Σ (nUsUo + nDsDo)/Σ (nUsDo + nDsUo) for candidates tested as positively co-regulated, and the inverse ratio, Σ (nUsDo + nDsUo)/Σ (nUsUo + nDsDo), for candidates tested as negatively co-regulated.
3. Supporting evidence from a minimum of three distinct experimental conditions (based on unique experimental condition codes).
4. Confirmation across a minimum of three independent publications (distinguished by PubMed ID).

Phosphosites satisfying all four criteria were designated high-confidence PsOPs and retained for all subsequent biological interpretation.

### 2.5 Co-occurrence analysis of SMG1 predominant phosphorylation sites

A co-occurrence analysis was conducted to investigate relationships between phosphorylation sites within SMG1 itself, focusing on the co-regulation patterns of phosphosite pairs (as distinct from the SMG1-to-other-protein PsOP analysis in Section 2.3). Differential datasets containing measurements for multiple SMG1 phosphosites under the same experimental conditions were selected for this analysis.

For each pair of SMG1 phosphosites, the frequencies of all four regulatory states, both sites upregulated (UU), one up and one down (UD), both downregulated (DD), and the reverse discordant state (DU), were quantified across the selected datasets. Positive co-regulation between a phosphosite pair was defined as the ratio: Σ (nUU + nDD)/Σ (nUD + nDU) and negative co-regulation as the inverse ratio: Σ (nUD + nDU)/Σ (nUU + nDD).

This approach enabled assessment of potential interdependence among SMG1 phosphorylation sites and identification of their functional proximity within co-regulatory networks. A heatmap illustrating phosphosite co-occurrence was generated using the Matplotlib and Seaborn libraries in Python (Han and Kwak, 2023). In this visualization, phosphosite pairs exhibiting positive or negative co-occurrence with a frequency greater than three were represented using red and violet gradients, respectively; phosphosite pairs with a co-occurrence frequency exceeding this threshold were considered functionally related or likely to share similar regulatory roles.

### 2.6 SMG1 interaction mapping and identification of regulatory kinases, substrates, and phosphatases

Experimentally documented protein–protein interaction partners of SMG1 were retrieved from multiple curated resources: HPRD (Keshava Prasad et al., 2009), BIND (Bader et al., 2003), BioGRID (Oughtred et al., 2021), ConsensusPathDB version 35 (accessed 22.05.2023; Kamburov and Herwig, 2022), CORUM (accessed 03.03.2023; Tsitsiridis et al., 2023), and RegPhos 2.0 (accessed 24.05.2023; Huang et al., 2014).

Experimentally validated upstream kinases and phosphatases, together with their corresponding phosphosites linked to SMG1, were compiled from PhosphoSitePlus (accessed 22 May 2023; Hornbeck et al., 2015), Phospho. ELM 9.0 (accessed 24 May 2023; Dinkel et al., 2011), and RegPhos 2.0 (accessed 24 May 2023; Huang et al., 2014).

In parallel, computationally predicted upstream kinases and substrates of SMG1 were obtained using *in silico* prediction tools, including NetworKIN (predicted 4 January 2023; Linding et al., 2008), AKID (predicted 24 May 2023; Parca et al., 2019), and iKiP-DB (Mari et al., 2022). Because several of these tools require individual protein sequences as query input, the complete set of RefSeq protein sequences required by these prediction tools was processed programmatically via API support.

Additionally, kinases and phosphatases associated with SMG1 phosphosites identified through synthetic peptide screening by Johnson et al. (2023) and through functional site annotation by Ochoa et al. (2020) were incorporated, using a 90th-percentile threshold for inclusion.

### 2.7 Motif analysis of substrates of SMG1

Identification of phosphorylation motifs recognized by a given kinase enables prediction of additional candidate phosphosites and substrates, providing insight into kinase specificity, signaling network regulation, and prospective therapeutic targets (Poll et al., 2024). UPF1 (T28, S1084, S1089, S1107, and S1127) and TP53 (S15), two well-established substrates of SMG1, contain the characteristic [S/T]-Q substrate recognition motif, as reported in PhosphoSitePlus.

Building on this established motif, substrates associated with the predominant SMG1 phosphosites identified through the FET-based co-regulation analysis (Section 2.3) were subsequently subjected to motif analysis using PhosphoSitePlus and manual comparison to assess the extent to which co-regulated candidate substrates share this canonical SMG1 recognition motif.

### 2.8 Data visualization tools

Lollipop plots depicting phosphosite frequency and distribution were generated using trackViewer, an R/Bioconductor package (10.18129/B9. bioc.trackViewer), in R. g: Profiler (Raudvere et al., 2019) was used for gene set/biological process enrichment analysis. The distribution of phosphosites across datasets was illustrated using Pandas and Matplotlib in Python.

**FIGURE 1:**
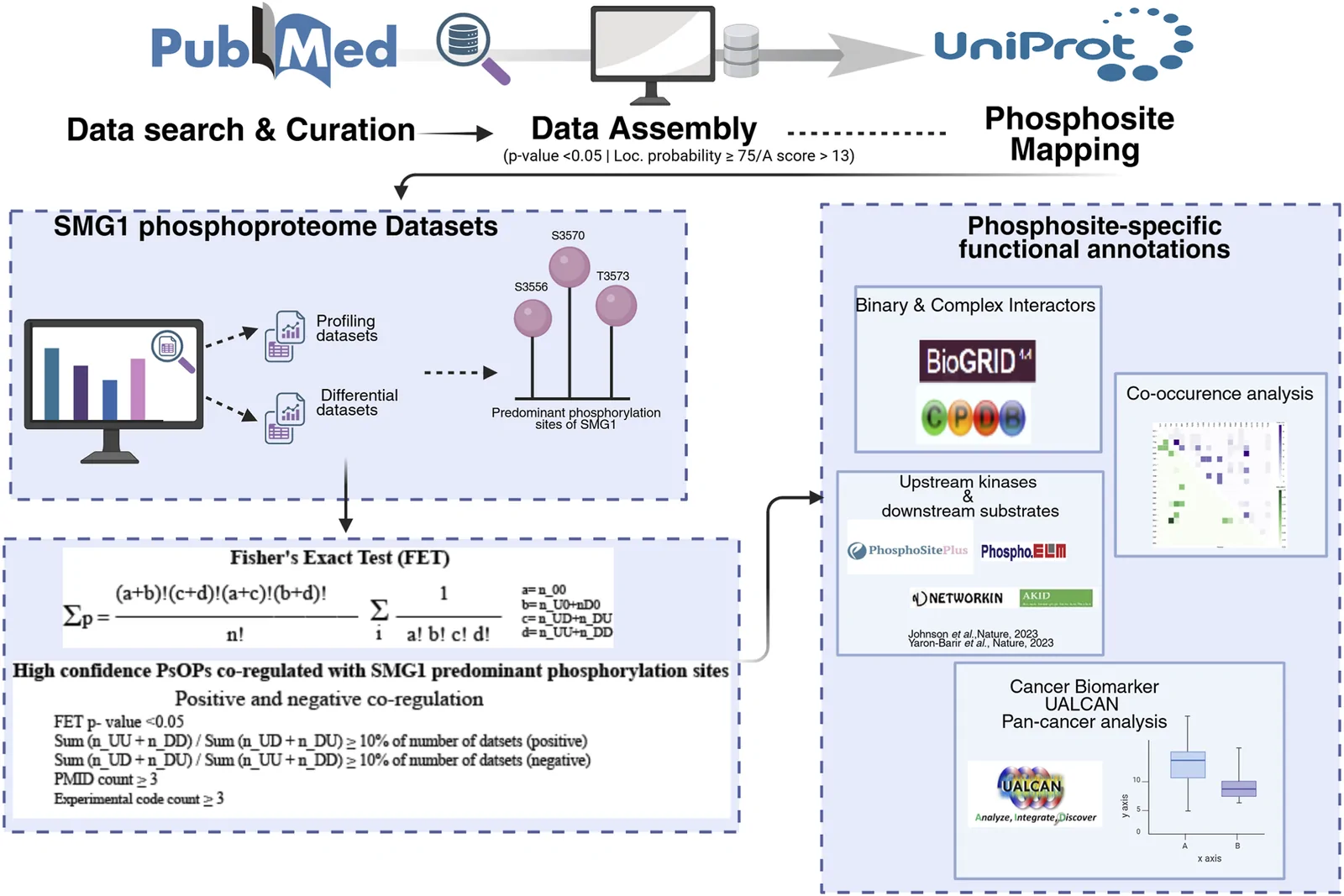
Integrated bioinformatics workflow for identifying SMG1-regulated phosphosites and their functional context.

Protein interaction networks were rendered using Cytoscape (version 3.10.3; Su et al., 2014), PathVisio 3 (Kutmon et al., 2015), and RAWGraphs 2.0 (https://www.rawgraphs.io/). The overall methodological workflow followed in this study is depicted in Figure 1.

## 3 Results

### 3.1 Global cellular phosphoproteome datasets containing class-I phosphosites and detection of predominant phosphorylation sites in SMG1

According to PhosphoSitePlus, SMG1 harbors 77 documented phosphorylation sites (Hornbeck et al., 2015); however, the biological roles and regulatory impact of these modifications remain essentially unexplored. To address this gap, we systematically curated and re-analyzed a large collection of publicly available phosphoproteomics datasets retrieved from PubMed, prioritizing high-confidence Class I phosphosites. Systematic screening, integration, and curation of phosphoproteomics data from more than 3,800 PubMed-indexed studies resulted in the compilation of mass spectrometry-derived human cell line phosphoproteomics datasets representing a broad range of experimental conditions. In total, SMG1 phosphosite data were extracted from 678 global phosphoproteome profiling studies and 173 differential phosphoproteomics experiments, spanning a wide range of cell types, perturbations, and time points (Supplementary Tables S1, S2). This exhaustive resource provides the foundation for subsequent analyses of SMG1 phosphorylation dynamics and regulatory networks. An overview of the compiled SMG1 phosphoproteomic profiling and differential datasets is provided in Table 1.

**TABLE 1:** Summary of the human cellular SMG1 phosphoproteomic datasets included in this study.

Characteristic	Profiling datasets	Differential datasets
Number of records	678	173
Unique PMIDs	126	49
Mapped SMG1 phosphosites	65	18
Unique experimental conditions	678	173
Main quantification type	SILAC (175), label-free (184), TMT (232), isotope labeling (12), dimethyl labeling (13), mTRAQ (33)	SILAC (59), mTRAQ (4), iTRAQ (1), label-free (27), TMT (53), dimethyl labeling (17)
Main enrichment methods	TiO2 (315), IMAC (137), peptide immunoaffinity enrichment (11), Fe- IMAQ (64)	TiO2 (116), IMAC (21), Fe- NTA (16), PY1000+PY100 (2), pTyr1000 (5), PolyMAC-Ti (5)
Most represented sites	T3573 (89), S3570 (70), S3556 (66), T3569 (38), T3550 (28), S3576 (18), and S34 (12)	T3573 (89), S3570 (70), S3556 (66)

**FIGURE 2:**
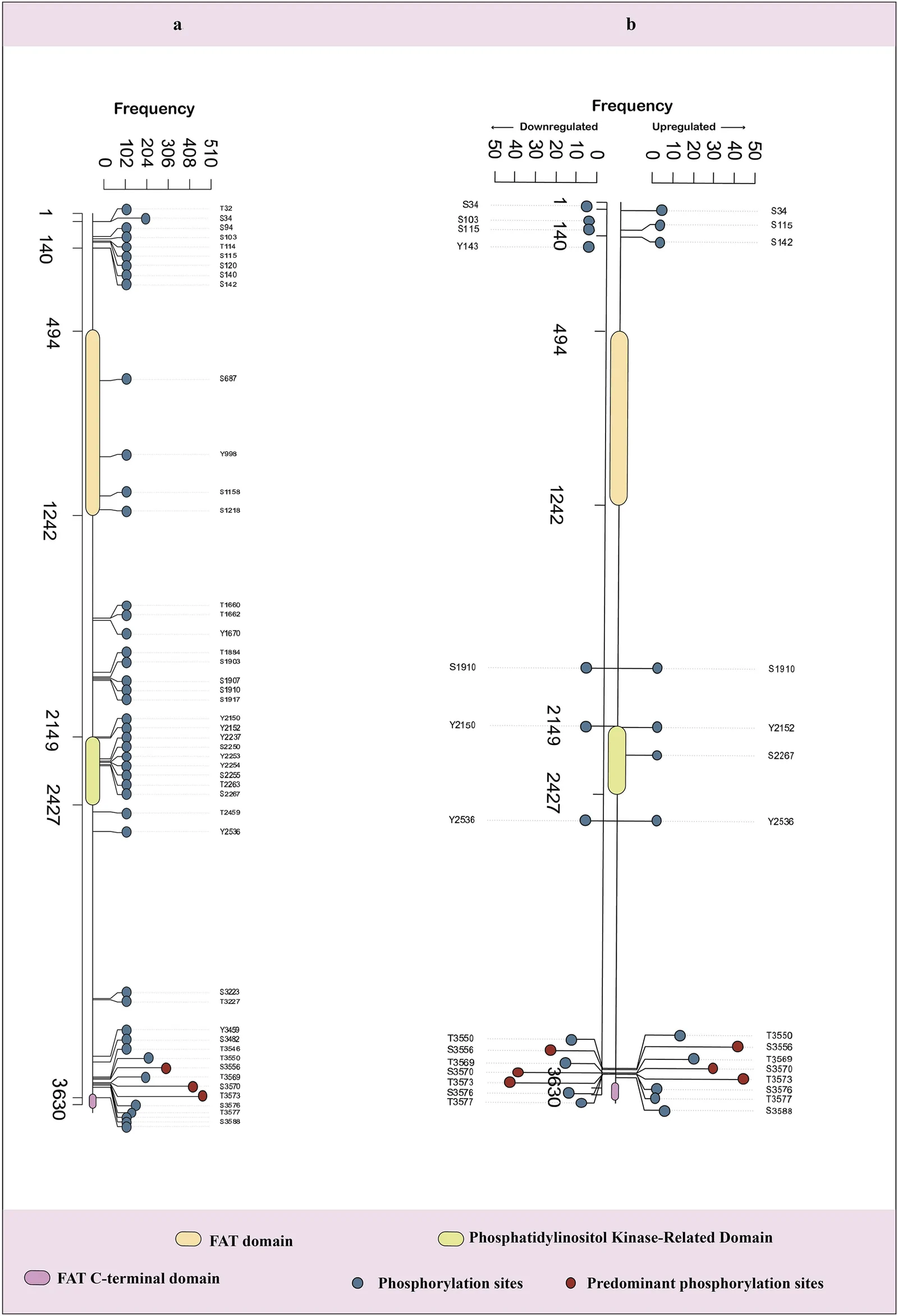
Lollipop plot illustrating the frequency distribution of SMG1 phosphorylation sites from phosphoproteome profiling and differential regulation datasets. **(a)** Class 1 phosphorylation sites detected in SMG1 across 678 profiling datasets. **(b)** Class 1 phosphorylation sites showing differential regulation in SMG1 across 173 quantitative differential datasets.

The differential data included expression patterns of 18 phosphosites, whereas the profiling datasets documented phosphorylation at 65 phosphosites in SMG1. Among these, T3573, S3570, and S3556 were the most abundantly detected sites, with respective counts of 509, 445, and 286 in profiling datasets, and 89, 70, and 66 in differential datasets. Notably, SMG1 (T3573) and SMG1 (S3570) were detected on the same tryptic peptide in the C-terminal region. Figure 2 shows the frequency of SMG1 phosphorylation sites found in the global cellular phosphoproteomics datasets.

### 3.2 Co-regulation analysis of predominant SMG1 phosphorylation sites

To gain deeper insights into the regulation of SMG1 and its role in cellular processes, we performed a coregulation analysis to examine phosphosites in other proteins (PsOPs) that consistently exhibit co-differential regulation with the predominant phosphorylation sites of SMG1. For each differential dataset, phosphosites were initially classified and ranked according to their upregulation or downregulation patterns relative to the expression status of the predominant SMG1 phosphorylation sites. 349 positively co-regulated and nine negatively co-regulated high confidence PsOPs were identified with SMG1 (T3573), and 470 positively co-regulated and 53 negatively co-regulated high confidence PsOPs were identified with SMG1 (S3570), and 917 positively co-regulated and 19 negatively co-regulated high confidence PsOPs were identified with SMG1 (S3556) (Supplementary Tables S3–S6).

**FIGURE 3:**
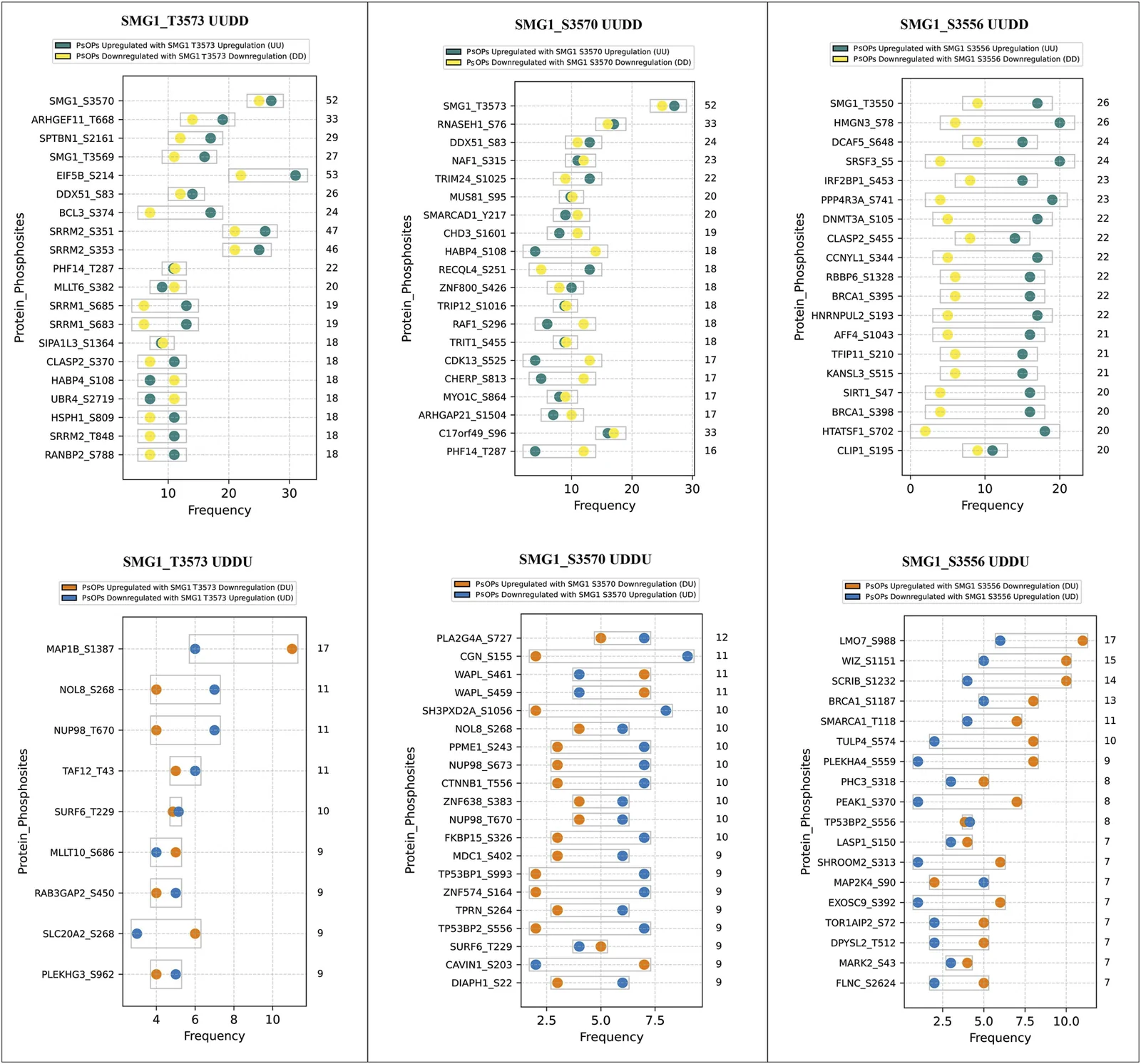
Dot plot representing the top 20 high-confidence protein phosphosite co-regulations with SMG1 predominant phosphorylation sites T3573, S3570, and S3556.

To facilitate interpretation across phosphosites that differ in their overall detection frequency, co-regulation strength is expressed here as the percentage of studies in which co-regulation was observed, in addition to the absolute dataset frequency. The top positively co-regulated PsOPs of SMG1 (T3573) were Rho guanine nucleotide exchange factor 11 (ARHGEF11) (T668) and Spectrin beta chain, non-erythrocytic 1 (SPTBN1) (S2161), with positive co-regulation frequencies of 33 and 29, respectively (observed in 37% and 32% of studies in which both phosphosites were detected), and the top negatively co-regulated PsOPs of SMG1 (T3573) were Microtubule-associated protein 1B (MAP1B) (S1387) and Nucleolar protein 8 (NOL8) (S268), with negative co-regulation frequencies of 17 and 11, respectively (observed in 37% and 32% of studies in which both phosphosites were detected). The top positively co-regulated PsOPs of SMG1 (S3570) were Ribonuclease H1 (RNASEH1) (S76) and ATP-dependent RNA helicase DDX51 (DDX51) (S83), with positive co-regulation frequencies of 33 and 24, respectively (observed in 47% and 34% of studies in which both phosphosites were detected), and the top negatively co-regulated PsOPs were Cytosolic phospholipase A2 (PLA2G4A) (S727) and Cingulin (CGN) (S155), with negative co-regulation frequencies of 12 and 11, respectively (observed in 17% and 15% of studies in which both phosphosites were detected). Similarly, the top positively co-regulated PsOPs of SMG1 (S3556) were High mobility group nucleosome-binding domain-containing protein 3 (HMGN3) (S78) and DDB1- and CUL4-associated factor 5 (DCAF5) (S648), with positive co-regulation frequencies of 26 and 24, respectively (observed in 39% and 36% of studies in which both phosphosites were detected), and the top negatively co-regulated PsOPs of SMG1 (S3556) were LIM domain only protein 7 (LMO7) (S988) and Protein WIZ (WIZ) (S1151), with negative co-regulation frequencies of 17 and 15, respectively (observed in 25% and 22% of studies in which both phosphosites were detected). Figure 3 shows the top 20 positively and negatively co-regulated PsOPs associated with predominant phosphorylation sites of SMG1.

### 3.3 Co-occurrence analysis of SMG1 predominant phosphorylation sites

**FIGURE 4:**
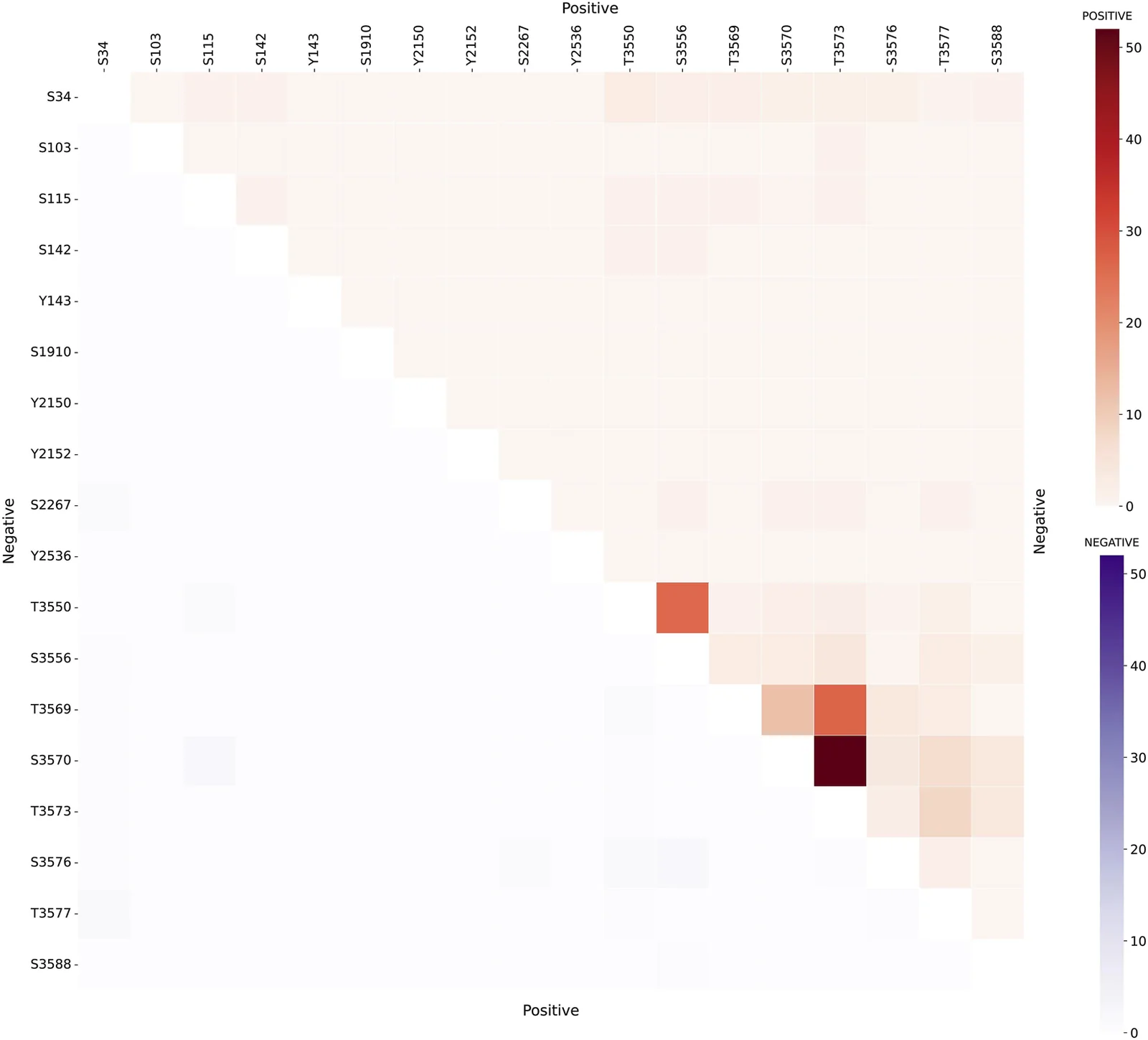
Co-occurrence analysis of SMG1 phosphosites based on co-regulated expression patterns. The heatmap illustrates relationships between phosphosite pairs, with red indicating positive co-regulation and violet indicating negative co-regulation, while the color intensity reflects the frequency of these co-regulation events.

Phosphorylation sites that frequently co-occur are often associated with functional similarity and tend to be evolutionarily conserved (Li et al., 2017), suggesting that they may collectively contribute to specific biological functions, subcellular localization, or molecular interactions (Sheela et al., 2026). To investigate this in the context of SMG1, we analyzed the co-occurrence patterns of differential regulation among its phosphosites. Notably, the predominant phosphosite S3556 showed strong co-occurrence with T3550 across 26 differential datasets and T3573 showed positive co-occurrence with T3569 and S3570 in 27 and 52 datasets respectively, indicating potential site-level coordination within the SMG1 phosphorylation landscape. The observed co-regulatory relationships among SMG1 phosphosites are illustrated in Figure 4, with detailed co-occurrence data provided in Supplementary Table S18.

### 3.4 Binary interactors of SMG1 involved in nonsense-mediated mRNA decay

**FIGURE 5:**
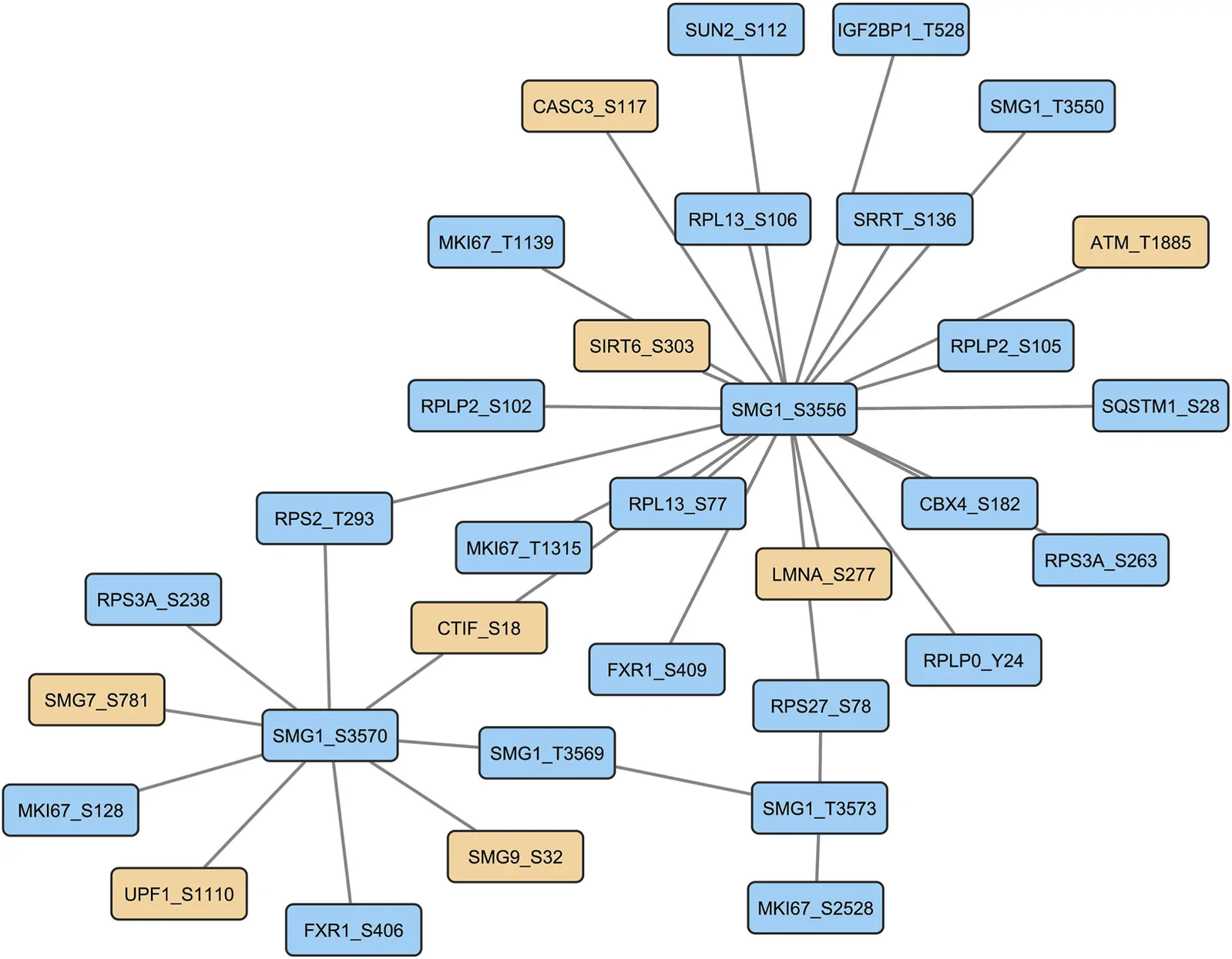
Binary interactors of SMG1at predominant phosphorylation sites with orange colour indicating the PsOPs involved in NMD.

To investigate the relationship between co-regulated phosphosites and the predominant phosphorylation sites of SMG1, we assembled a list of proteins documented to interact with SMG1 from various repositories. Positive co-regulation was observed for 4, 10, and 22 high-confidence binary interactors with phosphorylation sites T3573, S3570, and S3556, respectively, whereas no high-confidence interactors exhibited negative co-regulation at these predominant SMG1 phosphorylation sites. SMG1 is a kinase that plays a crucial role in nonsense-mediated mRNA decay (NMD) by directly phosphorylating UPF1, a central NMD factor, thereby triggering the degradation of mRNAs harboring premature termination codons (PTCs) (Lloyd et al., 2018). Several proteins directly involved in the nonsense-mediated mRNA decay (NMD) pathway were found to be positively co-regulated with predominant SMG1 phosphorylation sites. Specifically, Nonsense-mediated mRNA decay factor SMG7 (SMG7) (S781), Regulator of nonsense transcripts 1 (UPF1) (S1110), and Nonsense-mediated mRNA decay factor SMG9 (SMG9) (S32) were positively co-regulated with the phosphorylation site SMG1 (S3570). Protein CASC3 (CASC3) (S117) showed positive co-regulation with SMG1 (S3556), and CBP80/20-dependent translation initiation factor (CTIF) (S18) was uniquely co-regulated with both SMG1 (S3570) and (S3556) (Figure 5). However, the functional significance of most of these co-regulated binary interactors at specific phosphorylation sites remains largely unknown.

SMG7 plays a central role in nonsense-mediated mRNA decay (NMD) by linking the NMD machinery to mRNA degradation, interacting with UPF1 and SMG5 through its N-terminal domain and promoting transcript decay via its C-terminal domain (Okada-Katsuhata et al., 2012). SMG9 is a key component of the nonsense-mediated mRNA decay (NMD) pathway that forms a regulatory complex with SMG1, supporting its kinase activity and contributing to the efficient degradation of aberrant transcripts (Lai et al., 2024). CASC3 functions as a peripheral component of the exon junction complex (EJC) that promotes nonsense-mediated mRNA decay (NMD) by enhancing the degradation of EJC-dependent transcripts through endo-nucleolytic cleavage at the termination codon (Gerbracht et al., 2020). CTIF contributes to nonsense-mediated mRNA decay (NMD) by facilitating the targeting of misfolded polypeptides produced as NMD byproducts to the aggresome, thereby connecting NMD with cellular protein quality control mechanisms (Park et al., 2020). The list of binary interactors is given in Supplementary Table S13.

### 3.5 Putative upstream kinases of SMG1 S3556

While PhosphositePlus has catalogued 77 phosphorylation sites on SMG1, the kinases driving these modifications and their functional relevance are mostly uncharacterized. Phosphomotif-based prediction tools, including the approach developed by Johnson et al., have nonetheless nominated candidate kinases likely to target specific SMG1 phosphosites. To evaluate the reliability of these predictions, we used co-regulation analysis, comparing phosphorylation levels of predicted upstream kinases with those of corresponding SMG1 phosphosites, as a confidence filter to identify potentially coordinated signaling relationships.

**FIGURE 6:**
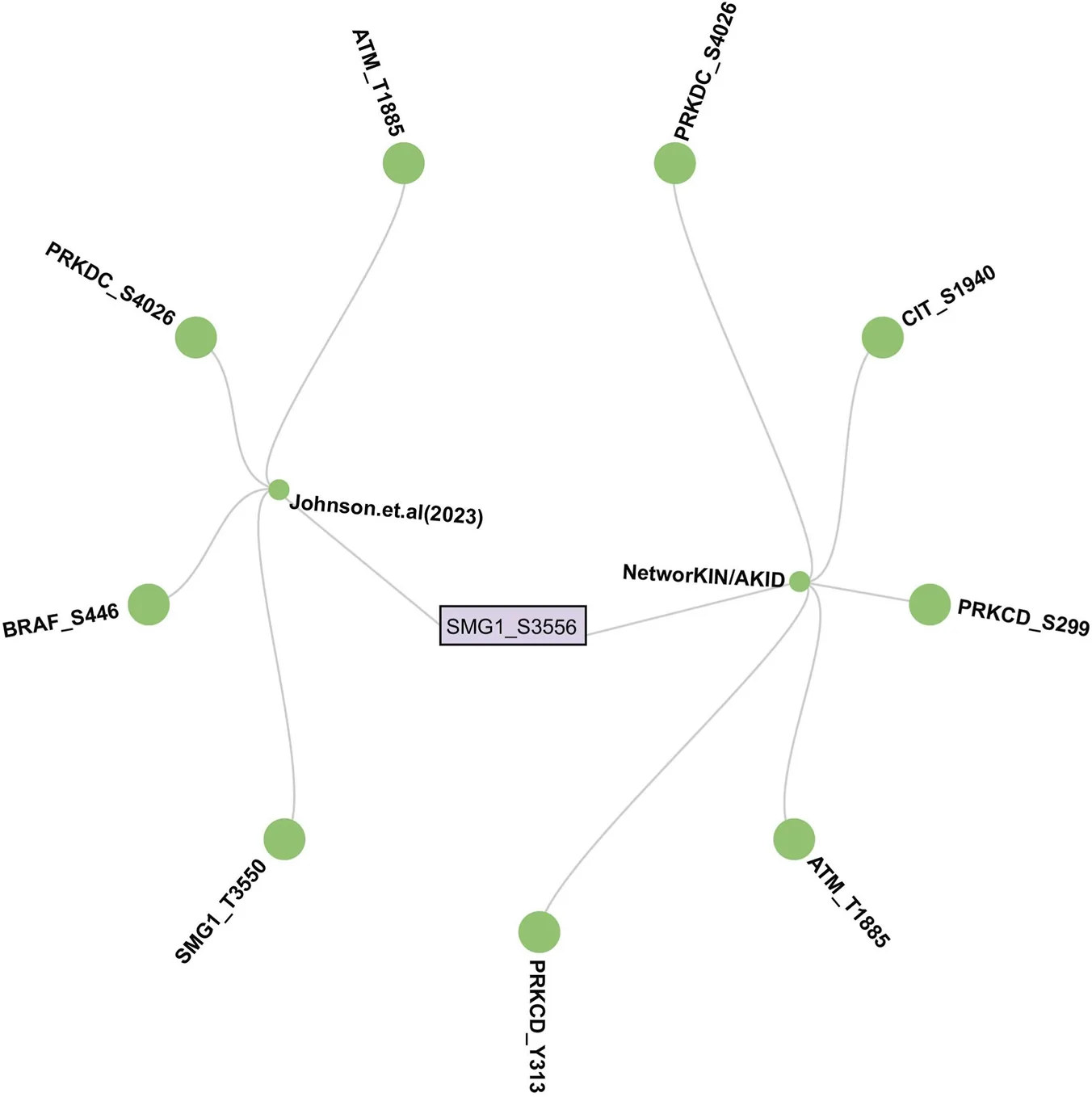
Representation of predicted kinases from Johnson et al. and NetworKIN/AKID showing positive co-regulation with SMG1 S3556.

This analysis revealed positive co-regulation between several predicted kinase phosphosites and SMG1 S3556. The candidate kinases showing this co-regulatory pattern were identified through two complementary prediction frameworks: Johnson et al. and NetworKIN/AKID. Of these, PRKCD (S299, Y313) and CIT (S1940) were exclusive to NetworKIN/AKID predictions, while SMG1 (T3550) and BRAF (S446) emerged solely from the Johnson et al. approach. PRKDC (S4026) and ATM (T1885) stood out as being independently nominated by both methods, lending greater confidence to their predicted roles. In contrast, no upstream kinases could be assigned to the two most prominent SMG1 phosphosites, T3573 and S3570, under the criteria applied. The full list of positively co-regulated kinases from the Johnson et al. framework is provided in Supplementary Table S14 and depicted in Figure 6.

BRAF is a serine/threonine kinase in the MAPK pathway that controls cell growth, differentiation, and survival (Toye et al., 2024) and is widely used as a target in cancers (Gouda and Subbiah, 2023; Tokuyama et al., 2024). PRKDC encodes the DNA-dependent protein kinase catalytic subunit (DNA-PKcs), the core component of the DNA-PK complex, which senses DNA damage and facilitates repair through both non-homologous end joining (NHEJ) and homologous recombination (HR) pathways (Bonczek et al., 2022). ATM and SMG1 participate in a DNA damage-induced alternative splicing pathway that regulates TP53 splicing and modulates radiation-induced cellular senescence (McCann et al., 2023). CIT, a serine/threonine kinase essential for cytokinesis as a key midbody component, is often overexpressed in tumors and promotes cancer cell proliferation (Sahin et al., 2019).

### 3.6 Motif analysis of SMG1 substrates linked to the DNA damage response (DDR)

SMG1 is an atypical kinase with only two experimentally validated substrates reported in PhosphoSitePlus: UPF1 (T28, S1084, S1089, S1107, S1127) and TP53 (S15), both of which contain the characteristic substrate recognition motif [S/T]-Q. Co-regulation analysis of SMG1 predominant phosphorylation sites T3573, S3570, and S3556 identified 21, 34, and 61 positively co-regulated substrates, respectively, while 1, 4, and three substrates were found to be negatively co-regulated at these sites. The list of predicted upstream kinases and substrates, along with their phosphosite co-regulation data, is provided in the Supplementary Tables S13,S14.

**FIGURE 7:**
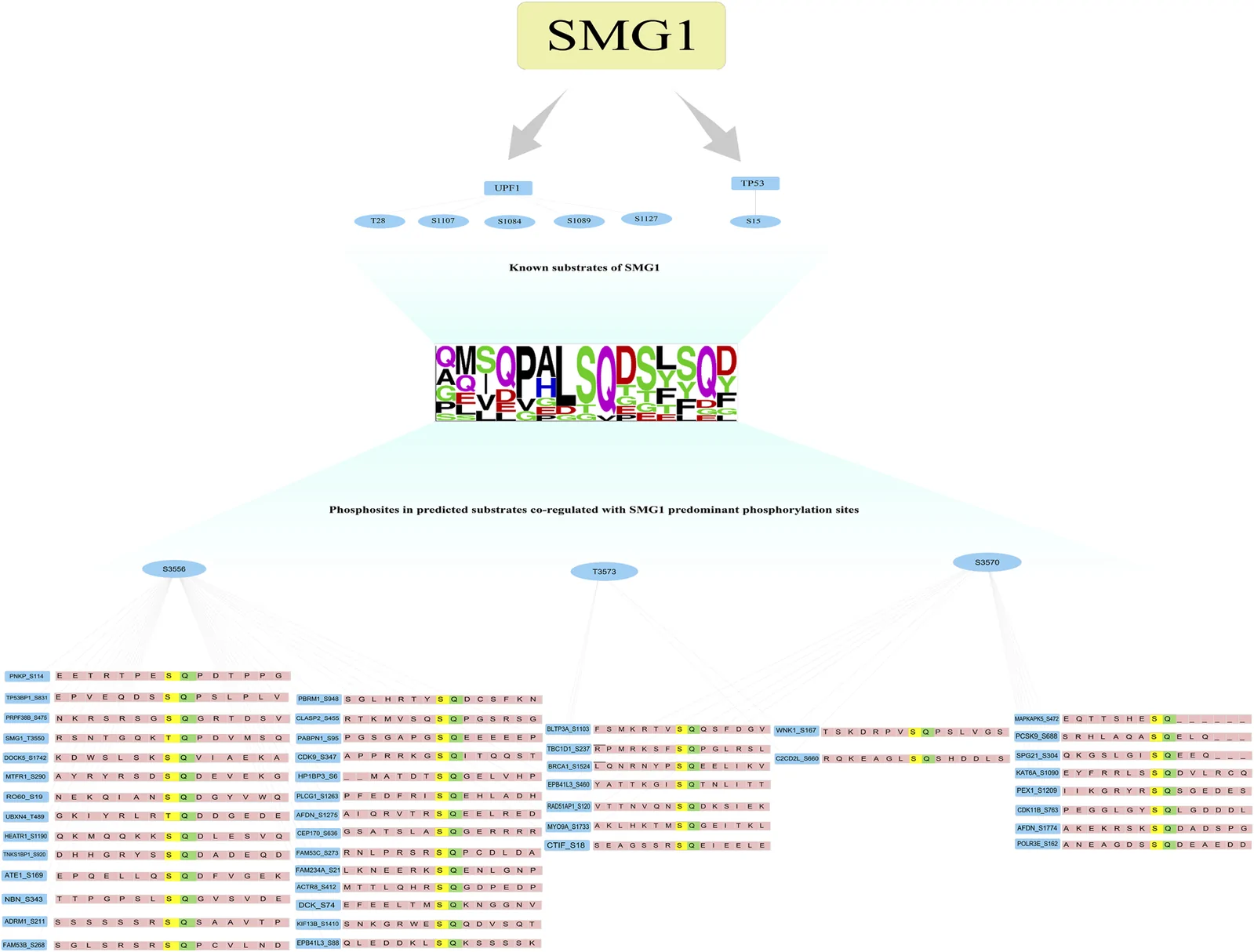
Representation of SMG1 substrates with S/T-Q motif.

**FIGURE 8:**
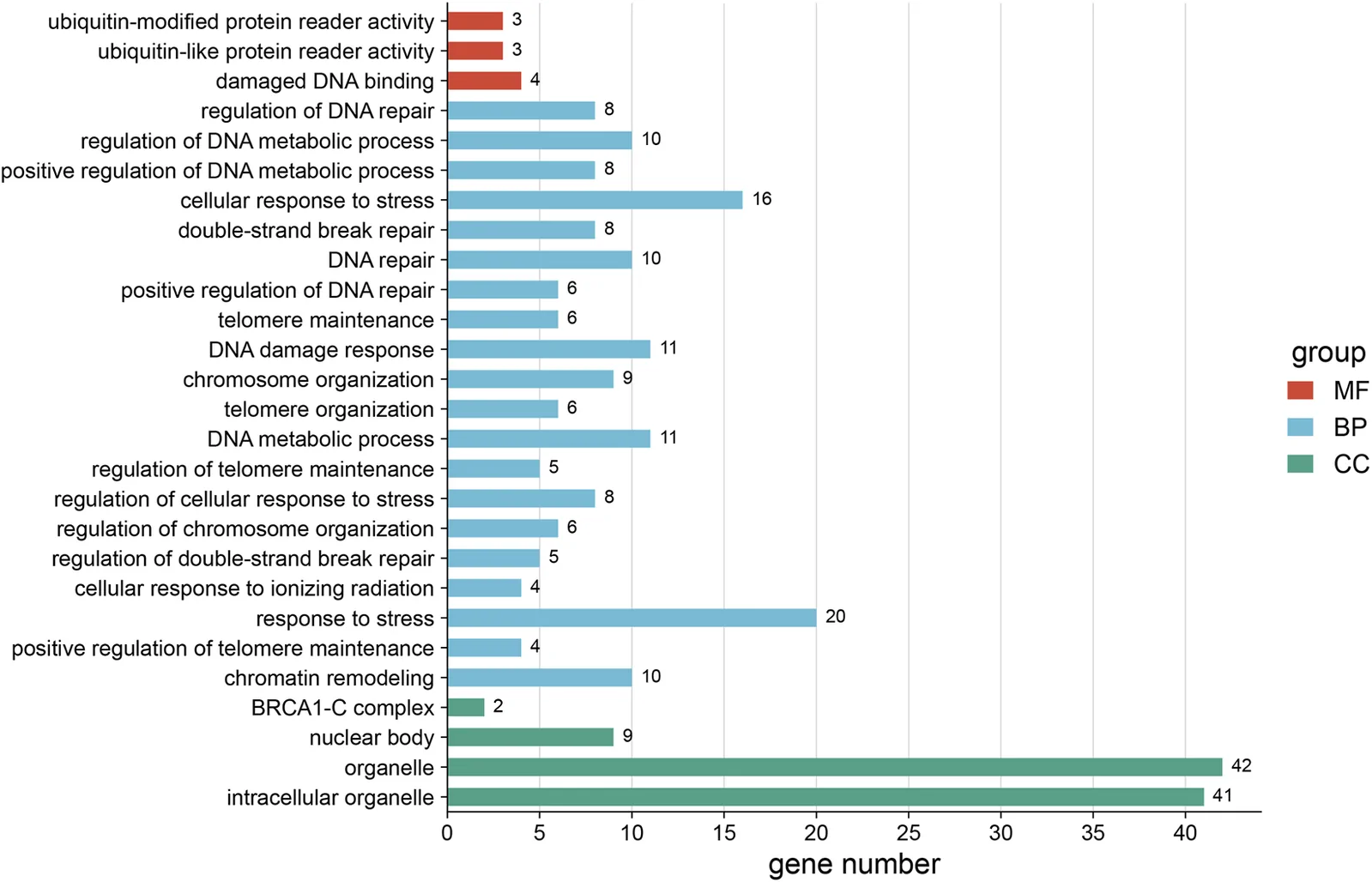
Gene enrichment of SMG1 substrates containing the [S/T]-Q phosphorylation motif. Enriched GO terms are categorized into Biological Process (BP), Molecular Function (MF), and Cellular Component (CC). The x-axis represents the number of genes associated with each enriched term, highlighting the involvement of SMG1 PsOPs in DNA damage response, DNA repair, chromatin remodeling, stress response, and nuclear cellular compartments.

Gene enrichment analysis of substrates having these [S/T]-Q motif (Figure 7) was further analyzed using g: Profiler, which revealed that SMG1 phosphorylates multiple substrates involved in the DNA damage response. The dot plot displays significantly enriched Gene Ontology biological process terms associated with these substrates (Figure 8). Breast cancer type 1 susceptibility protein (BRCA1) (S1524), RAD51-associated protein 1 (RAD51AP1) (S120), Serine/threonine-protein kinase SMG1 (SMG1) (T3550), 182 kDa tankyrase-1-binding protein (TNKS1BP1) (S920), Actin-related protein 8 (ACTR8) (S412), Cyclin-dependent kinase 9 (CDK9) (S347), Nibrin (NBN) (S343), Bifunctional polynucleotide phosphatase/kinase (PNKP) (S114), TP53-binding protein 1 (TP53BP1) (S831), Protein polybromo-1 (PBRM1) (S948). Details of the gene enrichment analysis of substrates are provided in (Supplementary Tables S7–S12). Mutations in BRCA1 disrupt its DNA-damage–induced complex with BCLAF1 and mRNA-splicing factors, impairing DDR-dependent splicing of repair genes and leading to defective repair and genomic instability (Savage et al., 2014). TNKS1BP1 has recently emerged as a key factor in double-strand break repair, offering stronger mechanistic support for developing anticancer strategies that inhibit PARP-1 and DNA-PKcs (Zou et al., 2015). ACTR8, a core subunit of the INO80 complex, supports its ATPase function and recruitment to DNA damage sites, and its loss phenocopies INO80 deficiency in DNA replication, repair, recombination, transcription, and heterochromatin maintenance (Choe et al., 2020). CDK9–cyclin K complex was discovered when cyclin K was identified as a p53-responsive transcriptional target activated during DNA damage (Anshabo et al., 2021). NBN functions in the early DNA damage response as part of the MRN complex with MRE11 and RAD50, sensing double-strand breaks and recruiting key repair factors such as ATM, H2AX, and BRCA1-containing complexes to initiate DNA repair (Belhadj et al., 2023). Polynucleotide kinase/phosphatase (PNKP) is essential for repairing DNA strand breaks by restoring 5′-phosphate and 3′-hydroxyl ends, and it supports multiple repair pathways through its interactions with proteins such as XRCC1 and XRCC4 (Weinfeld et al., 2011). 53BP1 is a central DDR mediator that guides DSB repair pathway choice through its multiple interaction interfaces, and its dysregulated expression is well linked to tumor development (Mirza-Aghazadeh-Attari et al., 2019). PBRM1 acts as a tumor suppressor, and when mutated it heightens cellular sensitivity to DNA-damage–based therapies, highlighting a potential therapeutic vulnerability in cancers such as ccRCC (Gu et al., 2022). Visualization of substrates having S/T-Q motif is represented in Figure 7.

### 3.7 SMG1 in cancers

**FIGURE 9:**
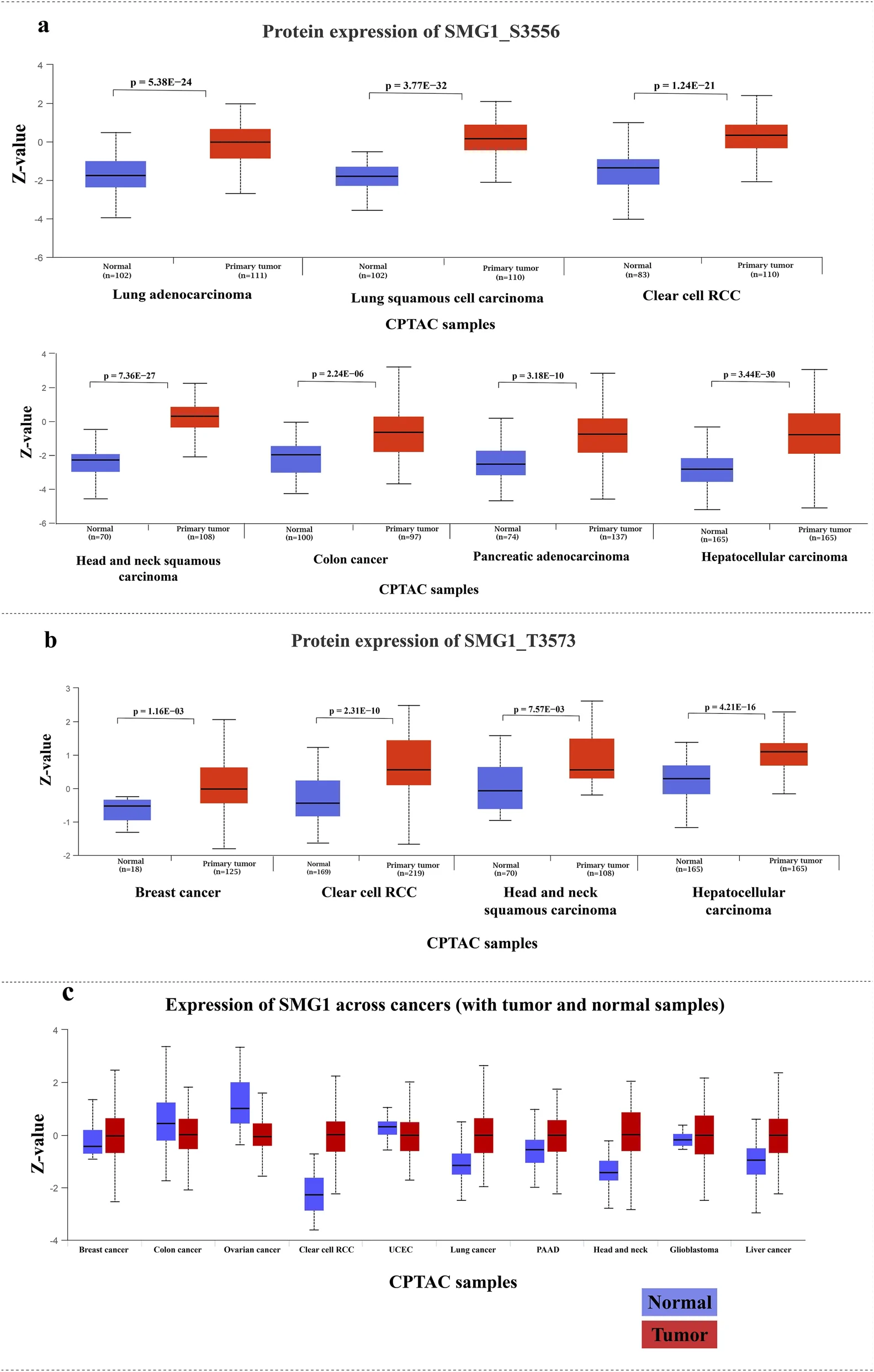
**(a,b)** Box plot illustrating the site-specific distribution of SMG1 across various cancer types. Blue boxes indicate normal tissue samples, while red boxes represent primary tumour samples for the respective cancers. **(c)** Pan-cancer analysis of SMG1 expression levels.

To explore the broader clinical implications of SMG1 in human malignancies, we analyzed proteogenomic data using the UALCAN web portal (Chandrashekar et al., 2022), a comprehensive platform for accessible analysis of publicly available datasets from TCGA and CPTAC consortia. Our analysis revealed that the predominant phosphorylation sites of SMG1 are differentially regulated across multiple cancer types (Figure 9). Z-score–based normalization was employed to quantify protein and phosphosite-level alterations, enabling robust comparison across heterogeneous tumor cohorts. Statistical significance was determined using an unpaired t-test (p < 0.05), as implemented in UALCAN. Critically, the patterns observed in Figure 9 are not uniform: certain cancer types show elevated phosphosite abundance relative to normal tissue, while others display reduced levels, and in some cases the direction of change differs between T3573/S3570 and S3556. This heterogeneity indicates that SMG1 phosphorylation is highly tumor-context-dependent, and that individual phosphosites may serve functionally distinct roles across different malignancies rather than acting as a single coherent signal.

To further investigate the functional relevance of these phosphorylation dynamics, we analyzed known and predicted SMG1 substrates in the context of cancer. A significant proportion of the substrates predicted by Johnson et al. are established regulators of genome stability and tumor suppression, including BRCA1, PBRM1, EPB41L3, TP53BP1, and NBN. Rather than representing isolated associations, the convergence of these substrates on DNA damage response and chromatin regulation pathways suggests that SMG1 may function as a central coordinator of genome integrity through motif-dependent phosphorylation. The tumor-context-dependent phosphosite alterations observed in Figure 9 thus carry a plausible functional consequence: dysregulation of individual phosphosites may selectively impair distinct arms of this substrate network, for instance, reduced S3556 activity in cancers where this site is downregulated could compromise ATM/DNA-PKcs-dependent signaling through BRCA1 and NBN, while altered T3573 or S3570 levels could disrupt cytoskeletal or RNA-processing co-regulatory networks, respectively.

It is important to note that the CPTAC-based analyses in Figure 9 capture phosphosite-level regulatory dynamics, a dimension of SMG1 biology that is mechanistically distinct from, and not captured by, measurements of total protein abundance. Prior expression-based studies have reported reduced SMG1 mRNA or protein levels across several malignancies, including acute and chronic myeloid leukemia (Karami et al., 2022; Hojjatipour et al., 2022), head and neck carcinoma (Zhang et al., 2020), hepatocellular carcinoma (Han et al., 2014), and pancreatic cancer (Wong et al., 2019). In those cancer types, transcriptional silencing or promoter hypermethylation drives overall loss of SMG1. However, the phosphosite alterations we document here represent an additional, post-translational layer of regulation that can operate independently of changes in total protein abundance. The integration of these findings suggests that SMG1 may be regulated at multiple levels in cancer, where changes in phosphosite activity can occur alongside or independently of alterations in total protein abundance. This distinction highlights the importance of phosphoproteomic analyses in capturing regulatory dynamics that are not apparent from expression data alone.

At the systems level, the SMG1 substrate network is enriched for proteins involved in DNA double-strand break repair, chromatin remodeling, and p53-dependent signaling pathways (Stefansson and Esteller, 2012; Squatrito et al., 2012; Escherich et al., 2025; Liu et al., 2025). Rather than acting through isolated tumor suppressors, these substrates form an interconnected functional module, suggesting that SMG1-dependent phosphorylation may coordinate multiple components of the DNA damage response simultaneously. In this context, dysregulation of SMG1 phosphorylation observed in our analysis (Figure 9) could disrupt coordinated signaling across these pathways, thereby contributing to impaired genome maintenance in cancer in ways that would be invisible to expression-level analyses alone.

Collectively, these observations position SMG1 not merely as an individual tumor suppressor-associated kinase but as a central regulator within a broader network governing genome stability. The combined evidence from phosphosite-level alterations (Figure 9) and prior expression studies supports a model in which SMG1-mediated signaling is inactivated through multiple, non-exclusive mechanisms in cancer, transcriptional silencing that may eliminates the kinase in some tumour types, and phosphosite-specific dysregulation that selectively rewires its downstream effector networks in others, with potential implications for cancer progression and therapeutic targeting.

## 4 Discussion

Here, by interrogating more than 3,800 high-confidence phosphoproteomics studies, we provide the first global, phosphosite-resolved view of SMG1 regulation and uncover unexpected layers of coordination between NMD, DNA-damage signalling, and tumour suppression. The present work extends beyond site cataloguing reported in PhosphoSitePlus and integrates heterogeneous phosphoproteomics datasets to provide quantitative, reproducible cross-dataset analysis and phosphosite-resolved correlation network.

Remarkably, the three most prevalent and dynamically regulated phosphosites, T3573, S3570, and S3556, cluster within an unstructured ∼150-amino-acid tail immediately downstream of the FATC domain. This extreme C-terminal segment has never been resolved in cryo-EM reconstructions (Zhu et al., 2019), and yet it dominates the detectable phosphoproteome of a 3,661-residue protein. The enrichment of these sites, particularly the co-detection of T3573 and S3570 on the same peptide, implies that this tail functions as a previously unrecognized regulatory hub. Sequential or mutually exclusive modification of these closely spaced residues could generate a phosphorylation barcode that routes SMG1 activity toward distinct effector networks, cytoskeletal remodeling (T3573), RNA processing (S3570), or chromatin/DNA-damage responses (S3556), providing an elegant mechanism for context-dependent kinase specificity.

The strong positive co-regulation of core NMD factors (SMG7, UPF1, SMG9, CASC3, and CTIF) with S3570 and/or S3556 reveals that phosphorylation cascades do not stop at SMG1 to UPF1 but extend across the entire NMD machinery (Ohnishi et al., 2003; Okada-Katsuhata et al., 2012). This coordinated regulation likely amplifies pathway flux and ensures rapid, synchronous activation upon detection of aberrant transcripts. The complete absence of high-confidence negative co-regulation among binary interactors further suggests that NMD is predominantly controlled by feed-forward phosphorylation rather than opposing phosphatase activity.

A particularly intriguing finding is the placement of SMG1 within the canonical DNA-damage-response kinase hierarchy. In-silico and peptide-array predictions identify ATM and DNA-PKcs as upstream kinases of S3556, while downstream co-regulation networks are enriched for classical ATM/ATR substrates bearing [S/T]-Q motifs (BRCA1 S1524, NBN S343, TP53BP1 S831, PNKP S114, PBRM1 S948, etc.) (Matsuoka et al., 2007). Together with the known SMG1-p53(S15) axis (Gewandter et al., 2011), these data close a positive-feedback loop: DNA damage from ATM/DNA-PKcs to SMG1(S3556) and broader PIKK substrate phosphorylation and alternative TP53 splicing (Chen et al., 2017). This framework helps explain earlier observations that loss of SMG1 triggers spontaneous DNA damage and activates checkpoint signaling and also renders human cells more sensitive to ionizing radiation (Brumbaugh et al., 2004; Roberts et al., 2013).

The expanded substrate landscape also has immediate translational implications. Many newly implicated targets-BRCA1, PBRM1, TP53BP1, NBN, TNKS1BP1-are core components of homologous recombination, non-homologous end-joining, and alternative lengthening of telomeres (ALT) (Zhang, 2013; Daley and Sung, 2014; Tauchi et al., 2002; Chabanon et al., 2021; Zou et al., 2015). Epigenetic silencing of SMG1 may therefore induce a functional HR defect or ALT dependence even in BRCA1/2-proficient tumours, dramatically widening the patient population that could benefit from PARP inhibitors, ATR inhibitors, or radiotherapy (Lord and Ashworth, 2017). The rare but highly specific negative co-regulation of cytoskeletal and cell-junction proteins (MAP1B, LMO7, CGN) when SMG1 sites are phosphorylated further supports an anti-invasive role, consistent with clinical observations that SMG1 loss correlates with metastasis and poor prognosis (Han et al., 2014; Zhang et al., 2018).

Finally, the predicted regulation of S3556 by BRAF and the co-regulation of CDK9 place SMG1 at the convergence of multiple actionable signalling pathways (Yaeger and Corcoran, 2019). Combined inhibition of BRAF/MEK and SMG1 (or restoration of SMG1 function) could be explored in BRAFV600E melanomas, while CDK9 degraders might synergise with SMG1 modulation in MYC-driven malignancies.

**FIGURE 10:**
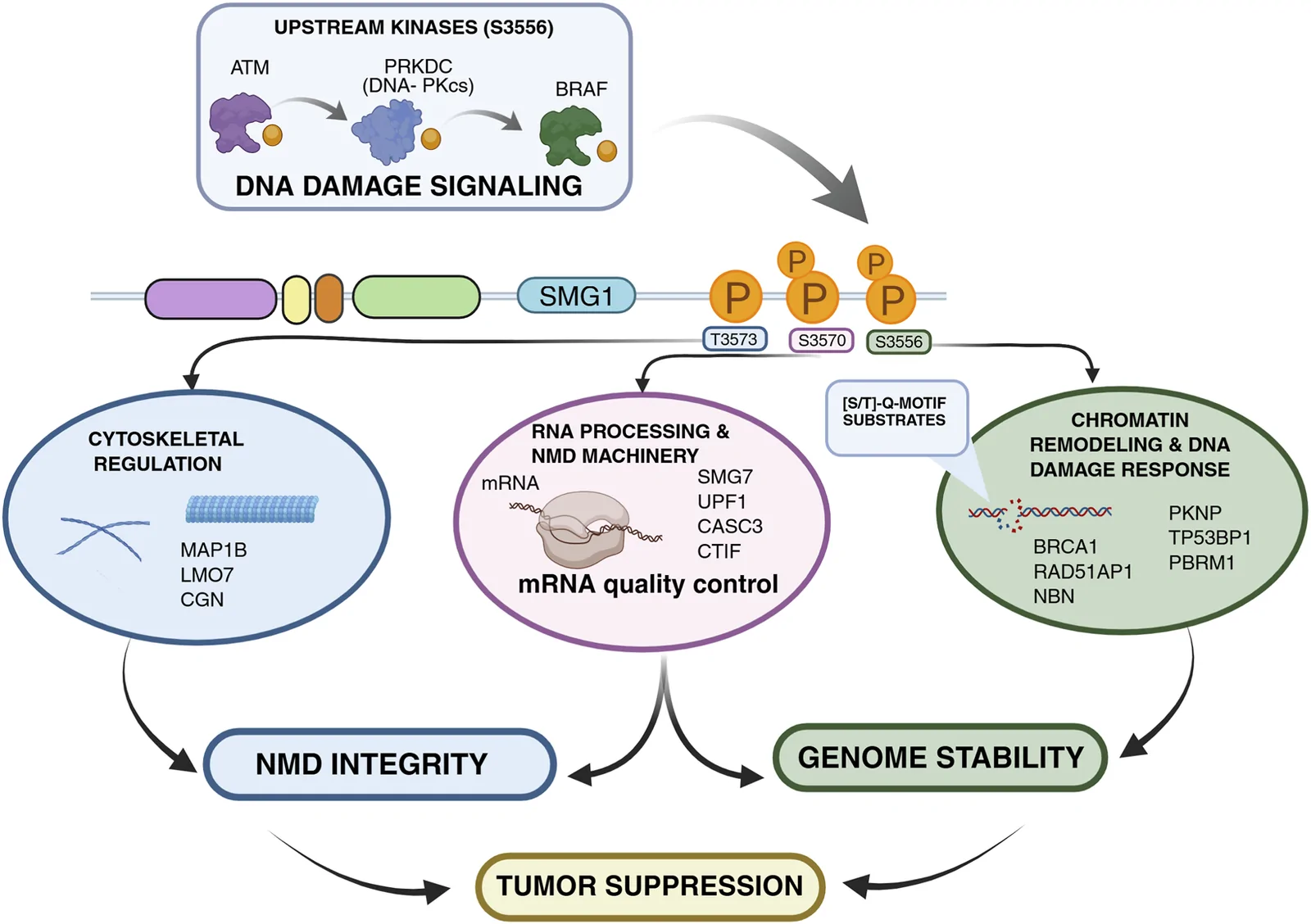
Model of SMG1 phosphorylation-mediated regulation of multiple cellular processes contributing to tumor suppression.

In summary, our phosphoproteomic meta-analysis repositions SMG1 from a specialised NMD kinase to a central phosphorylation hub that integrates mRNA surveillance with genome maintenance and tumour suppression (Figure 10). The identification of a C-terminal regulatory tail coordinated NMD phospho networks, an ATM–SMG1–DDR feedback loop, and an expanded tumor-suppressor substrate repertoire provides a rich framework for future mechanistic studies and opens new therapeutic avenues in cancers characterised by SMG1 inactivation or chronic genomic instability.

## 5 Limitations

Although this study establishes a novel phosphosite-centric framework for dissecting SMG1 regulatory networks, several limitations should be considered when interpreting and generalizing the results. First, the use of heterogeneous public phosphoproteomic datasets encompassing diverse platforms, experimental conditions, and cell types introduces unavoidable variability, despite stringent site-quality criteria (Class-1 sites with localization probability ≥75% and A-score ≥13) and multi-level filtering, and full normalization is limited by the lack of raw data and standardized reprocessing. Second, the correlation-based analytical strategy does not establish causal relationships, and the functional significance of many correlated phosphosites (PsOPs), particularly those that are poorly characterized, remains inferential without direct biochemical or functional validation. In addition, several established SMG1 regulatory phosphosites, including T3573, S3570, and S3556, were sparsely detected, likely to reflect inherent mass spectrometry challenges in capturing low-abundance or highly dynamic phosphorylation events. It should be also noted that the phosphoproteomic datasets used in this study were not specifically enriched for tyrosine phosphorylation sites. As pY sites are typically of low stoichiometry and require dedicated enrichment strategies for reliable detection, the limited representation of tyrosine phosphosites in our analysis likely reflects a technical bias rather than their true biological prevalence. Future studies incorporating pY-enriched datasets would provide a more comprehensive view of SMG1 tyrosine phosphorylation and its regulatory significance. Also, while our analysis focused on the three most frequently quantified SMG1 phosphosites, the biological relevance of the remaining sites cannot be excluded. The limited discussion of lower abundance phosphosites reflects constraints in data availability and detection frequency rather than an assessment of their functional significance, and future studies with broader phosphosite coverage are warranted. Moreover, although our analyses associate correlated phosphosites with specific biological processes, their direct functional roles in nonsense-mediated mRNA decay and the DNA damage response in cancer remain to be experimentally demonstrated. Overall, while our findings suggest previously unrecognized regulatory functions for these SMG1 phosphosites, comprehensive validation using time-resolved phosphoproteomics, phosphosite-specific mutagenesis, and functional assays will be necessary to define the precise architecture and biological significance of the SMG1 phosphorylation network.

## 6 Conclusions

This study represents the first comprehensive, phosphosite-centric investigation of SMG1 post-translational regulation, leveraging an unprecedented collection of 851 phosphoproteomics datasets. We establish T3573, S3570, and S3556 as the dominant and dynamically regulated phosphorylation sites on SMG1, each embedded in distinct functional networks: cytoskeletal dynamics, RNA metabolism, and chromatin/DNA-damage signaling, respectively. The robust positive co-regulation of key NMD effectors, SMG7, UPF1, SMG9, CASC3, and CTIF, with S3570 and S3556 reveals a previously unrecognized layer of coordinated phosphorylation control that likely fine-tunes the efficiency and specificity of nonsense-mediated mRNA decay. Furthermore, the identification of ATM, DNA-PKcs, and BRAF as candidate upstream kinases for S3556 integrates SMG1 into the canonical DNA-damage-response kinase hierarchy, whereas the discovery of numerous [S/T]-Q–containing DDR proteins (including BRCA1, NBN, PNKP, TP53BP1, and PBRM1) as putative downstream substrates substantially broadens SMG1’s functional scope beyond classical NMD.

Collectively, these findings reposition SMG1 as a multifunctional phosphorylation hub that bridges mRNA surveillance with genome stability maintenance, providing a molecular rationale for its frequent inactivation in diverse malignancies and its emerging status as a *bona fide* tumor suppressor. The regulatory map presented here offers a valuable resource for the community and paves the way for targeted functional studies, biomarker development, and therapeutic strategies aimed at restoring or modulating SMG1 activity in cancer and other diseases characterized by genomic instability or defective mRNA quality control.

## Data Availability

The original contributions presented in the study are included in the article/Supplementary Material, further inquiries can be directed to the corresponding authors.
